# Cement leakage following percutaneous kyphoplasty in a patient after a posterior lumbar fusion: a case report

**DOI:** 10.1186/s12893-020-00733-8

**Published:** 2020-04-15

**Authors:** Ziquan Li, Keyi Yu, Xiao Chang, Siyi Cai, Jun Gao, Yipeng Wang

**Affiliations:** 1 Department of Orthopedics, Peking Union Medical College Hospital, Chinese Academy of Medical Sciences, Shuaifuyuan No. 1, Wangfujing, Dongcheng District, Beijing, 100730 China; 2 Department of Neurosurgery, Peking Union Medical College Hospital, Chinese Academy of Medical Sciences, Shuaifuyuan No. 1, Wangfujing, Dongcheng District, Beijing, 100730 China

**Keywords:** Cement leakage, Percutaneous kyphoplasty, Postoperative lumbar fusion, Surgical removal

## Abstract

**Background:**

Percutaneous kyphoplasty (PKP) has become an important minimally invasive surgical technique for fracture stabilization and pain relief in patients with vertebral compression fractures. However, intraspinal cement leakage following PKP is a serious postoperative complication that can lead to morbidity and mortality.

**Case presentation:**

We describe an uncommon case of epidural leakage of bone cement in an 81-year-old woman who underwent posterior lumbar decompression and fusion from L3–5 4 years prior and had an unremarkable postoperative course. The patient was admitted to Peking Union Medical College Hospital with complaints of muscle weakness and severe low back pain radiating to the left thigh 1 week after PKP of L5 due to an acute osteoporotic compression fracture. Computed tomographic imaging revealed massive leakage of cement into the spinal canal at L5-S1, and therefore, surgical decompression and removal of epidural cement were performed carefully without causing a dural tear. She improved remarkably and no neurologic deterioration was observed in the postoperative period during the one-year follow-up.

**Conclusions:**

We present the rare reported case, to our knowledge, of epidural cement leakage after PKP at the segment of internal fixation and discuss the most likely etiologies and preventive measures for this condition.

## Background

Thoracolumbar vertebral compression fractures result in intractable low back pain, progressive spinal deformity, and neurological damage. It is widely recognized that percutaneous kyphoplasty (PKP) is a minimally invasive procedure performed to stabilize the vertebral fracture and alleviate pain with shorter postoperative recovery time [1]. However, PKP has the potential risk of several surgical complications, the most common of which is bone cement leakage. Although most cases are asymptomatic, some severe cases of cement leakage into the spinal canal, dura, or intervertebral foramen may cause neurological dysfunction or pulmonary embolism, and can be life-threatening [2–4]. To our knowledge, we present the rare reported case of an 81-year-old woman who suffered epidural cement leakage following PKP in the same vertebra with pedicle screws and clarify the most likely etiologies, clinical signs, and treatments of this rare condition.

## Case presentation

**Fig. 1:**
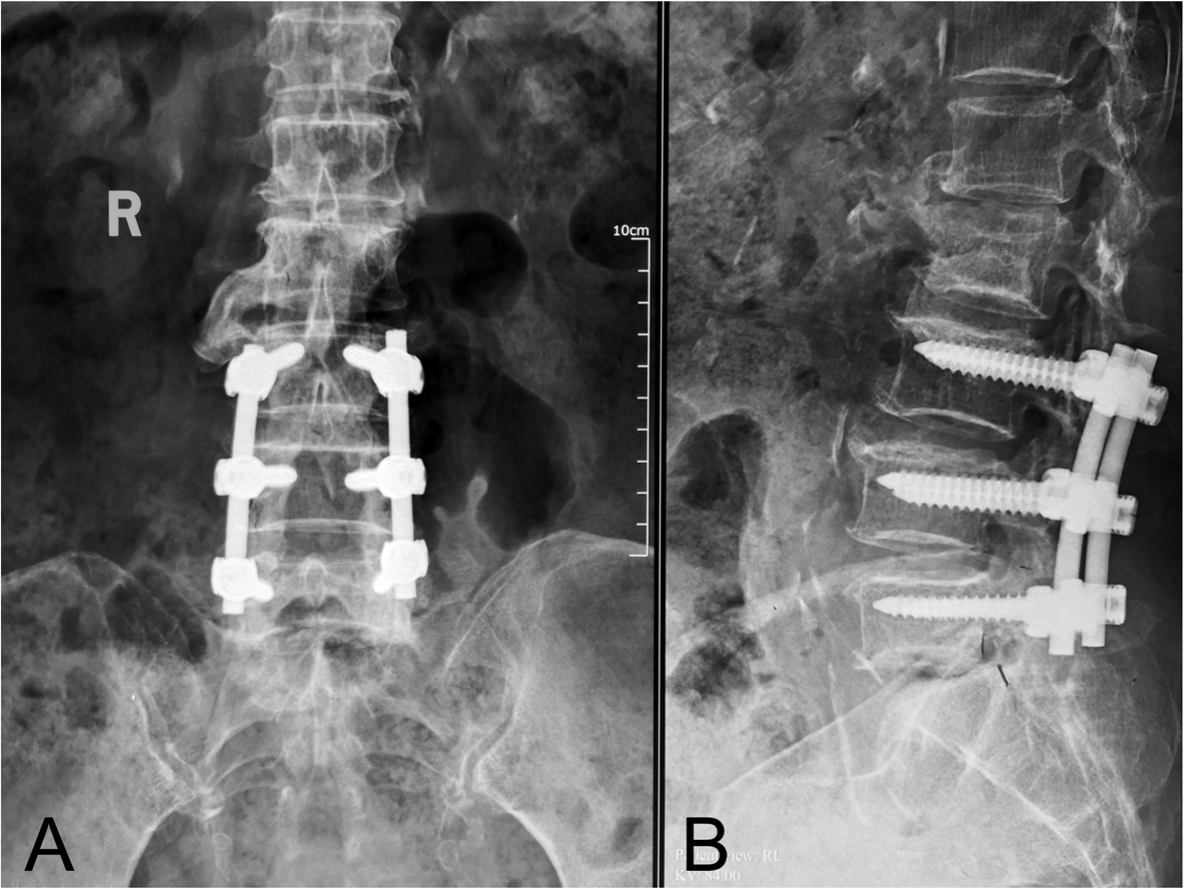
Anterior-posterior (AP) and lateral X-ray images of the lumbar spine demonstrated a vertebral compression fracture of L5 with good alignment of the construct from L3–5

A Chinese woman presented to Peking Union Medical College Hospital with complaints of low back pain and intermittent claudication. Based on physical examination and radiographic results, the diagnoses of lumbar spinal stenosis of L3–5 and lumbar spondylolisthesis of L4 were considered. She underwent posterior decompression, reduction of spondylolisthesis, internal fixation, and bone graft fusion from L3–5 without any postoperative complications. The patient’s initial recovery was uneventful, and her symptoms improved. Four years after the initial surgery, the patient was admitted to a local hospital for low back pain after a slip and fall. The possibility of acute vertebral compression fracture of L5 was considered (Fig. 1), and she underwent percutaneous kyphoplasty of L5 under local anesthesia.

**Fig. 2:**
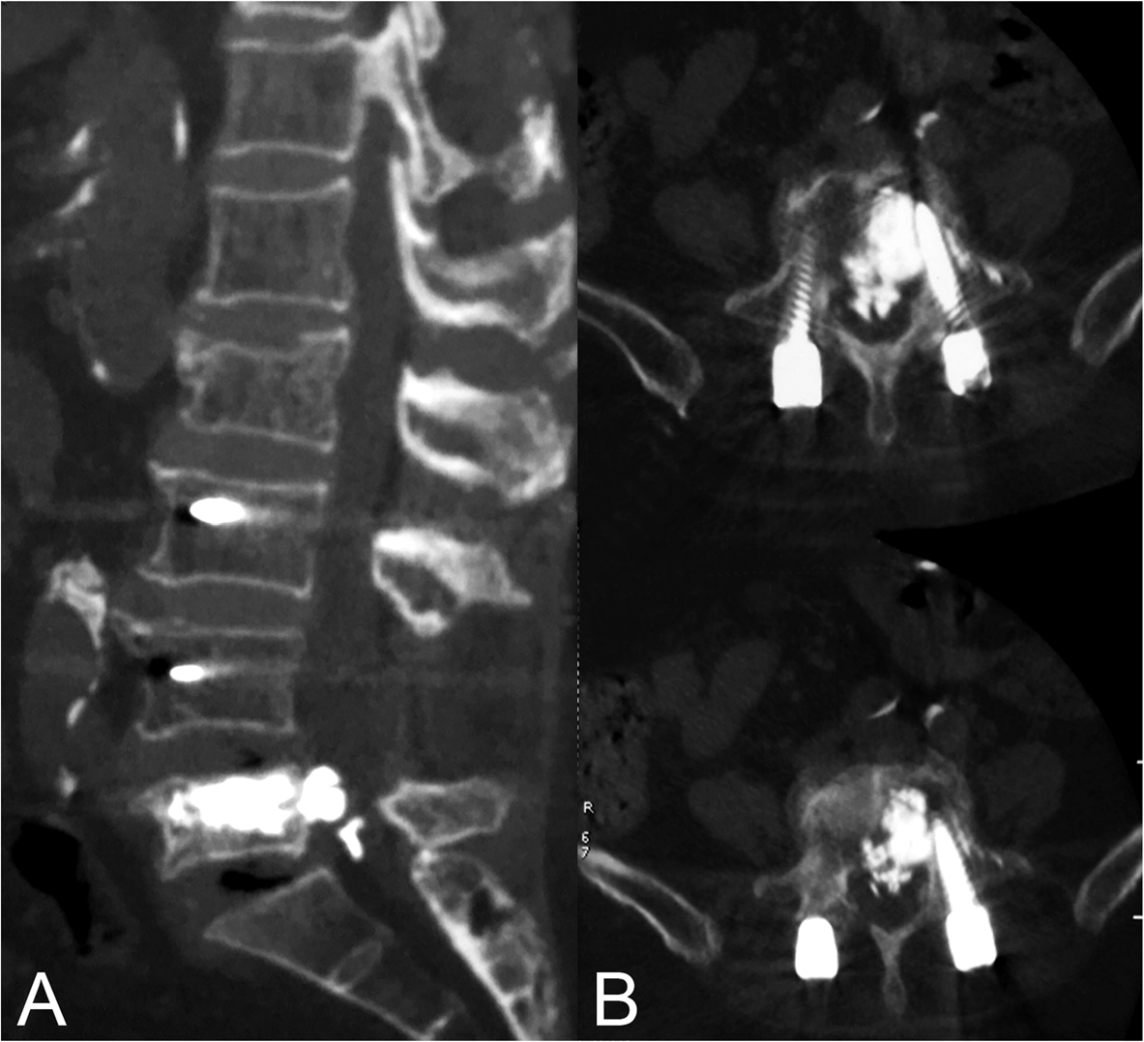
A massive epidural cement leakage at L5-S1 was illustrated in the sagittal (**a**) and axial (**b**) reconstruction planes of the computed tomography scan

The patient was hospitalized at our institution again due to the progression of low back pain with radiation to the left thigh 1 week after PKP surgery. The pain was severe and stabbing, and was aggravated by position changes. The numerical rating scale (NRS) scores for pain were 8 to 9 at the moment of severe pain. Physical examination revealed tenderness and percussion pain at the paravertebral and spinous processes of L5-S1 without restricted lumbar spine motion. Neurologic examination evaluated the muscle strength of left lower extremity as grade 4/5 according to the British Medical Research Council (BMRC) scale. Hyporeflexia of the Achilles tendon was detected on the left side, but pathologic reflexes, superficial reflexes, and the upper limb neurologic examination were unremarkable. Bowel and bladder function remained normal and there were no difficulty evacuating or urinary incontinence. The plain x-ray and CT scan revealed satisfactory position of the internal fixation but massive leakage of bone cement into the canal at the L5-S1 level, which resulted in significant compression of the S1 nerve root (Fig. 2).

**Fig. 3:**
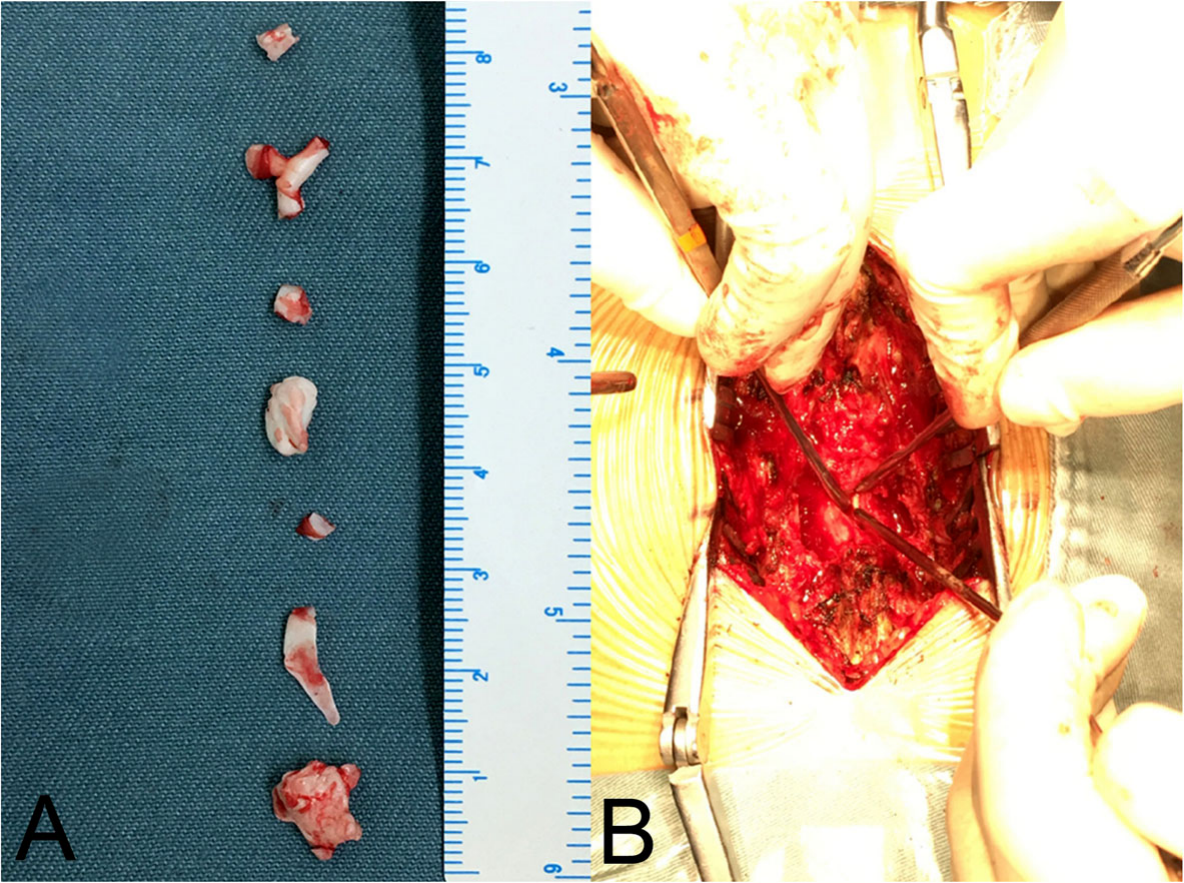
Intraoperative images showed that several pieces of leaked cement fragments had been removed (**a**) and bone cement was exposed after pulling the dural sac aside (**b**)

Based on the patient’s complaints, clinical signs, and radiological characteristics, a diagnosis of cement leakage following PKP was made. She then underwent posterior decompressive surgery. After resecting scar and granulation tissue from the initial surgery, the spinal cord was fully exposed and decompressed at L5-S1. While properly pulling the dural sac aside, epidural cement fragments, located in front of the spinal canal, were revealed and excised carefully without dural tear or any nerve root injury (Fig. 3).

**Fig. 4:**
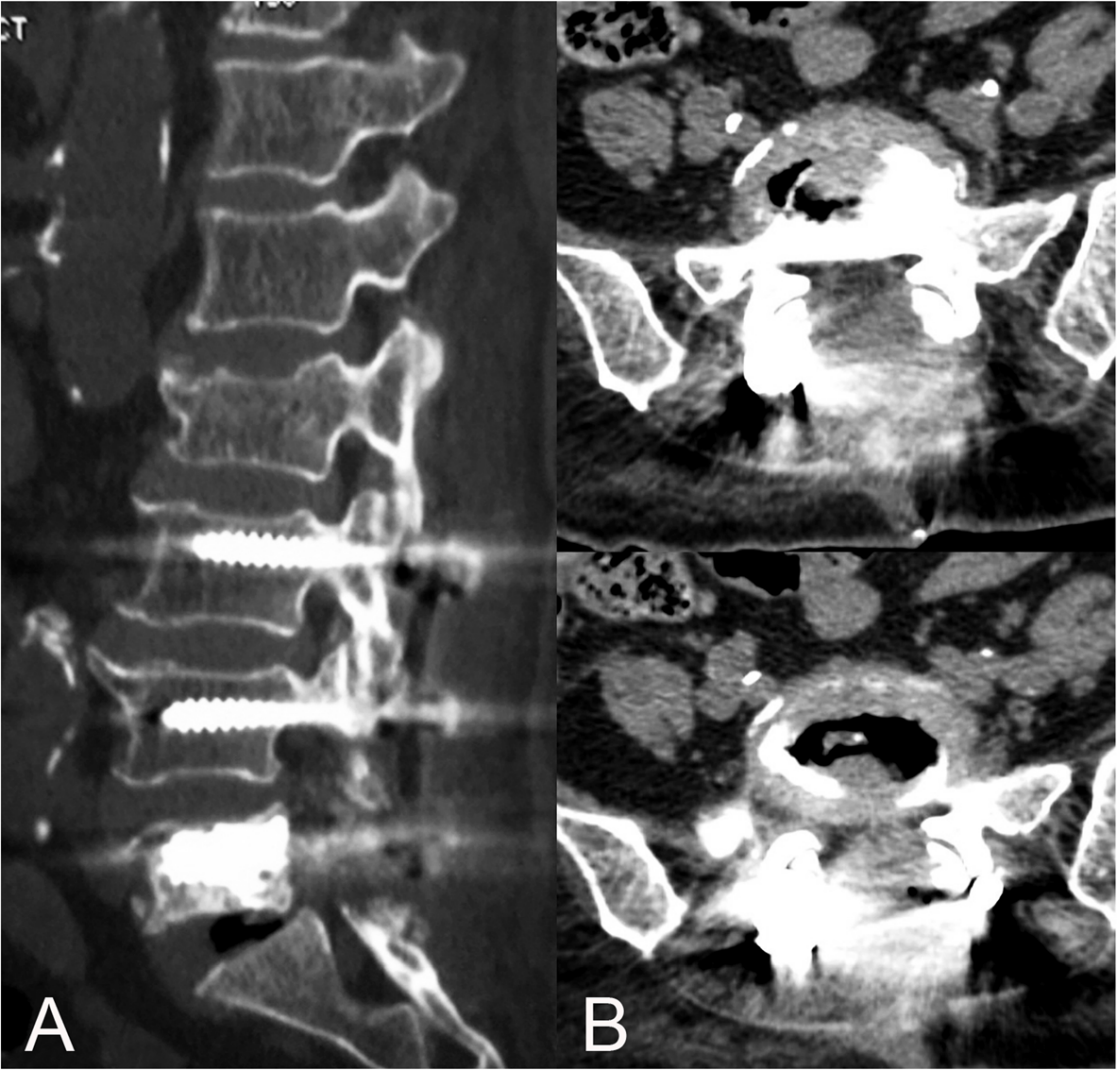
Postoperative sagittal (**a**) and axial (**b**) CT images demonstrated good alignment of the implant and the complete removal of epidural leaked cement with thorough decompression of the spinal cord at L5-S1

The radiating pain and neurologic deficit were immediately relieved after surgery, but the muscle weakness of the left lower extremity remained unchanged. Postoperative CT images indicated complete removal of the epidural cement (Fig. 4). The patient’s postoperative course was unremarkable and close follow-up was performed at 6 and 12 months postoperatively. No neurologic deterioration was observed in the postoperative period.

## Discussion and conclusions

Instrumented spinal fusion represents the most common surgeries for various spinal disorders; however, there have been only three documented case reports of compression vertebral fracture occurring within vertebra existing instrumentation or within the solid vertebral fusion [5–7]. In our case, osteoporotic compression fracture of instrumented vertebra was considered based on image examination, history of initial lumbar fusion surgery and history of falls. Decrease of vertebral body bone mineral density after spinal fusion surgery was demonstrated because of biomechanical stresses, postoperative immobilization and mineral metabolism [8]. Currently, percutaneous kyphoplasty and percutaneous vertebroplasty (PVP) surgery have become the standard treatments for osteoporotic vertebral compression fractures, and are effective for rapid pain relief, restoration of vertebral body height, and enhancing strength and rigidity of the involved vertebrae by minimal invasive injection of bone cement [9, 10]. However, as with any invasive procedure, complications related to percutaneous kyphoplasty are not uncommon, and include cement extravasation, pulmonary embolism, infection, epidural hematoma, systemic toxicity, and vertebral body fractures [11, 12]. Although the incidence of bone cement leakage following PKP surgery is significantly lower than that of PVP due to application of balloon dilation, cement leakage is still considered the most common complication of PKP [13].

At present, the incidence of bone cement leakage has varied between 5 and 87% in the literature [14, 15]. The most probable reason for the significant difference in the rate of bone cement leakage is that many studies mainly rely on x-ray examination for the diagnosis of bone cement leakage, rather than CT scan, which may result in missed diagnosis. Nieuwenhuijse et al. showed that the actual rate of bone cement leakage is higher than previously reported [14], and therefore, CT scans should be obtained to calculate the exact leakage rate and to assess persistent pain occurring postoperatively [16, 17]. Most clinical cement leakage following PKP requires no further treatment, so it is often overlooked clinically and is generally considered to be a normal phenomenon during surgery rather than a true complication. Nevertheless, when the bone cement leaks into the spinal canal or the intervertebral foramen, the spinal cord and the nerve root would be compressed to cause local or radicular pain, neurologic complications, and pulmonary embolization. Pain may be exacerbated when the cement leaks into the adjacent tissues or veins. Therefore, its potential harm is enormous and must be taken seriously in clinical practice.

Many factors contributing to the occurrence of bone cement leakage have been reported, including preoperative vertebral compression, viscosity of bone cement, the amount of injected bone cement, puncture approach, connection between the intravertebral cleft and basivertebral foramen, and preoperative verification of the fracture pattern (posterior wall or pedicle fracture) [18–21]. In our case, the compression fracture occurred in the L5 vertebral body with pedicle screws. Inserting the injector through pedicle with a screw or using an extrapedicular approach would be technically challenging. It was prone to penetrate into the spinal canal due to the obstruction of the pedicle screw when the PKP procedure was performed via the pedicle approach, thereby leading to cement leakage into the epidural space. The neural irritation symptoms due to epidural leakage could be related to the mass effect and secondary injury; to be more specific, the compression consequences of the spinal cord and nerve root were derived from cement extrusion into the spinal canal, and the exothermic reaction of the tissues during polymerization and cytotoxicity resulted from exposure of the tissues to residual bone cement. Considering our proposed risk factors, the following procedures may help prevent cement leakage following PKP: use of a bilateral transpedicular approach, a beveled needle, thicker consistency cement, and the avoidance of overfilling. It is critical to strictly follow the indications for surgery and complete preoperative imaging examinations, especially a CT scan, to identify the type of fracture. A PKP should be considered cautiously for patients with severe posterior wall vertebral injuries, the intravertebral cleft in vertebrae or compression fractures of the vertebrae with internal fixation. Intraoperative computed tomography-guided navigation and neuromonitoring might be useful tool in such complex cases.

These recommendations will help minimize the incidence of bone cement leakage after PKP. However, once cement leakage occurs, and is accompanied by a nerve deficit, the main goals of surgical intervention are to remove the bone cement, achieve spinal decompression, and relieve the secondary injury. As an alternative to conventional laminectomy, it has been reported that percutaneous endoscopic approach could achieve the targeted decompression with a smaller incision, decreased damage to soft tissues, and faster recovery. Moreover, the whole operation may be performed under local anesthesia, which allowed real-time neural function evaluation and had a lower risk of anesthesia-related complications [22]. Nevertheless, several limitations should be taken into consideration for minimal invasive surgery. Our patient underwent initial open surgery, which could be the challenge for percutaneous endoscopic interlaminar approach. On the other hand, transforaminal approach might be suffered from blockade of crista iliaca.

We report the rare case of intraspinal cement leakage after PKP at the level of the vertebrae with pedicle screws. It was prone to penetrate into the spinal canal due to the obstruction of the pedicle screw when the PKP procedure was performed, which reminded that PKP should be considered cautiously for compression fracture of vertebrae with internal fixation. Therefore, CT scans should be recommended to determine whether postoperative symptoms are related to cement extrusion. Immediate treatment for a neurologic deficit due to bone cement leakage is needed and the percutaneous endoscopic approach could be safely performed in patients with symptomatic cement leakage after a PKP procedure.

## Data Availability

All the data supporting our findings are contained within the manuscript.

## Abbreviations

PKP: Percutaneous kyphoplasty
NRS: Numerical rating scale
CT: Computed tomography
PVP: Percutaneous vertebroplasty
